# A Critical Analysis of the Available *In Vitro* and *Ex Vivo* Methods to Study Retinal Angiogenesis

**DOI:** 10.1155/2017/3034953

**Published:** 2017-08-07

**Authors:** A. F. Moleiro, G. Conceição, A. F. Leite-Moreira, A. Rocha-Sousa

**Affiliations:** ^1^Departamento de Cirurgia e Fisiologia, Faculdade de Medicina, Universidade do Porto, Porto, Portugal; ^2^Unidade de Investigação Cardiovascular, Faculdade de Medicina, Universidade do Porto, Porto, Portugal; ^3^Departamento de Cirurgia Cardiotorácica, Centro Hospitalar São João, Porto, Portugal; ^4^Departamento de Oftalmologia, Centro Hospitalar São João, Porto, Portugal

## Abstract

Angiogenesis is a biological process with a central role in retinal diseases. The choice of the ideal method to study angiogenesis, particularly in the retina, remains a problem. Angiogenesis can be assessed through *in vitro* and *in vivo* studies. In spite of inherent limitations, *in vitro* studies are faster, easier to perform and quantify, and typically less expensive and allow the study of isolated angiogenesis steps. We performed a systematic review of PubMed searching for original articles that applied *in vitro* or ex vivo angiogenic retinal assays until May 2017, presenting the available assays and discussing their applicability, advantages, and disadvantages. Most of the studies evaluated migration, proliferation, and tube formation of endothelial cells in response to inhibitory or stimulatory compounds. Other aspects of angiogenesis were studied by assessing cell permeability, adhesion, or apoptosis, as well as by implementing organotypic models of the retina. Emphasis is placed on how the methods are applied and how they can contribute to retinal angiogenesis comprehension. We also discuss how to choose the best cell culture to implement these methods. When applied together, *in vitro* and ex vivo studies constitute a powerful tool to improve retinal angiogenesis knowledge. This review provides support for researchers to better select the most suitable protocols in this field.

## 1. Introduction

Angiogenesis is a biological process in which new vessels are formed from previously established vessels [1]. In spite of being a physiological process, angiogenesis is also involved in several diseases.

The retina constitutes a tissue frequently affected by pathologic angiogenesis. Retinal vascular diseases, including diabetic retinopathy (DR), retinopathy of prematurity (ROP), and retinal vein occlusions, are diseases in which ischemia, leakage, and neovascularization from retinal vessels occur [2]. Both insufficient vascularization and excessive vessel formation may contribute to the disease's pathophysiology.

Neovascular retinal diseases have a tremendous effect on the quality of life. Therefore, it is important to understand their pathophysiology, as well as to find therapeutic agents able to interfere with their prognosis. Thus, appropriate methods should exist to recreate and to study the angiogenic process. A common problem in the study of angiogenesis, particularly in the retina, is the difficulty in the selection of the ideal method to apply. The perfect model should be not only physiologically reliable but also technically simple, inexpensive, and easy to accurately quantify.

Angiogenesis can be assessed through both *in vitro* and *in vivo* studies. *In vivo* studies may simulate angiogenesis more closely to reality. However, they are more complex to apply and more expensive and involve multiple cells and agents, which may hinder the evaluation of the intended effect [3].

On the other hand, *in vitro* studies can be criticized for being very different from the natural environment. However, *in vitro* studies have several advantages. First, they do not demand the technical skills in animal handling and have typically lower costs. Furthermore, *in vitro* assays allow the study of isolated steps that contribute to angiogenesis, permit the identification of specific effects on endothelial cell function besides being more rapid and easily quantified. Finally, *in vitro* studies have the advantage of allowing genetic manipulation of cells as well as the utilization of cells and tissues from transgenic species [3].

Organotypic cultures or ex vivo models maintain the architecture of the tissues closer to the *in vivo* setting, overtaking some of the limitations of the *in vitro* studies [4]. Additionally, they are not so technically exigent to implement as *in vivo* studies.

Therefore, both *in vitro* and ex vivo studies can be valuable to study the angiogenic process in the retina.

We present a systematic review of the available *in vitro* and ex vivo assays for retinal angiogenesis study, highlighting the following: (1) the cell lines and primary cultures of endothelial cells (ECs) used or the ex vivo models applied; (2) the sequential events of angiogenesis and the most appropriate methods to study the several aspects of the process; and (3) the applicability, advantages, limitations, and disadvantages of each method.

This is a review with interest that can help researchers better select the most suitable protocols in this field.

To improve the understanding of all the concepts of the work, we first present a brief synopsis of the angiogenic process.

### 1.1. Angiogenic Process

**Figure 1:**
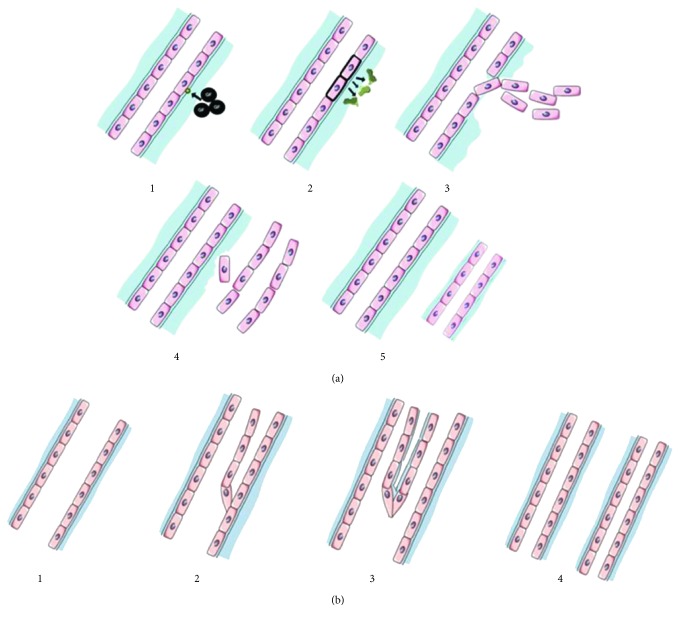
Types of angiogenesis. (a) Sprouting angiogenesis. (1) Endothelial cells are activated by growth factors. (2) Activated endothelial cells release proteases that degrade extracellular matrix. (3) Endothelial cells migrate and proliferate. (4) Endothelial cells start to maturate into a new vessel. (5) Stabilization of the new vessel. (b) Intussusceptive angiogenesis. (1) Stable vessel. (2) Extension of a preexisting vessel wall into the lumen. (3) The new vessel wall proliferates and splits the preexisting vessel. (4) Stabilization of the new vessels.

Angiogenesis is the process in which new vessels are formed from previously established vessels. There are two types of angiogenesis described: sprouting and nonsprouting or intussusceptive angiogenesis [1]. In sprouting angiogenesis, ECs in a preexisting vessel are activated by growth factors (GF) and then secrete proteases to degrade the preexisting vessel basement membrane. Thus, a breakdown arises in the vessel basement membrane, allowing activated ECs to migrate and proliferate. The process is initiated by a “tip cell” that leads the sprouting process and the “stalk cells” follow forming a chord. As the vessel maturation occurs, the stalk cells form a lumen and synthetize a surrounding basement membrane. The newly formed vessels are then surrounded by pericytes and stabilized, maturing into capillaries [5]. After the primary vascular plexus is formed, more ECs are generated, producing new capillaries by sprouting or by splitting from their vessel of origin [1]. In the other type of angiogenesis, intussusceptive angiogenesis, the vessel wall of a preexisting vessel extends into the lumen, dividing it into two [1, 5] (Figure 1).

Similarly to the brain, the predominant mechanism of retinal vascularization is sprouting angiogenesis, although additional modes of vascular growth such as intussusception are also present. Actually, intussusceptive angiogenesis may constitute a mechanism of vascular adaptation to hypoxia [6]. Even vasculogenesis, in which vessels are formed by concatenation of vascular precursor cells into solid cords that then lumenize, might contribute to retinal vascularization, although existing evidence is still controversial [7].

The type of angiogenesis in each tissue depends on the number of vessels already present, when it starts to grow rapidly [1]. Angiogenic response differs spatially and temporally, since the angiogenic environment also varies, presenting distinctive characteristics within different organs, ages, and physiological or pathologic conditions [1].

In physiological conditions, angiogenesis is a highly regulated process that results from complex interactions between GF which stimulate or inhibit ECs [8]. For its occurrence, first, GF need to activate ECs. Several inducers of angiogenesis have been identified, including the members of the vascular endothelial growth factor (VEGF) family, angiopoietins, transforming growth factor (TGF), platelet-derived growth factor (PDGF), tumor necrosis factor-*α* (TNF-*α*), interleukins (IL), and the members of the fibroblast growth factor (FGF) family. Furthermore, some other factors control and influence angiogenesis including soluble GF, such as cytokines, membrane-bound proteins and lipids, extracellular matrix (ECM) components, and cell-cell interactions, including fibroblasts, pericytes, and smooth muscle cells (SMCs) [9, 10].

In retinal ischemic diseases, new blood vessels grow from the preexisting retinal vasculature. Indeed, in proliferative retinal diseases, VEGF, produced in response to hypoxia by retinal pigment epithelium (RPE) cells, plays a central role increasing vascular permeability and promoting neovascularization [11]. Moreover, under hypoxic conditions, inflammatory responses can potentiate neovascularization. Several proinflammatory cytokines may induce blood vessel formation via direct engagement of target ECs or, indirectly, by inducing leukocytes and/or ECs to produce proangiogenic mediators [8]. The blood vessel formation constitutes an attempt to repair ischemia. However, vessels are unable to grow within the retina towards the ischemic retina, due to the lack of suitable ECM to support their growth. Therefore, the new vessels break through the basement membrane of the retina, causing hemorrhage and scar tissue contraction that contribute to additional damage [5]. Additionally, some angiogenic factors may elicit proinflammatory responses in ECs by upregulating the expression of cell adhesion molecules and inflammatory mediators [8]. RPE cells are a critical element in factors' secretion, modulating the angiogenic and inflammatory responses [11, 12].

Whatever the type or the condition in which angiogenesis occurs, ECs need to escape quiescence, proliferate, migrate, and undergo tubulogenesis to form functional vessels. Thus, the angiogenic process can be defined as occurring in a number of sequential events: angiogenic sprouting, vessel formation, adaptation to tissue needs, and stabilization [10]. These sequential processes constitute the basis of angiogenesis *in vitro* assays.

## 2. Research Design and Methods

In order to achieve this systematic review, specific criteria were defined. First, two queries related to the object were built to search on PubMed. The first query was intended to find papers analyzing *in vitro* methods: “Angiogenesis” [All fields] AND (“in vitro” [All fields] OR “cell culture” [All fields] OR “microfluidic system” [All fields]) AND (“retinal endothelial cells” [All fields] OR “choroidal endothelial cells” [All fields]). The second query was intended to find papers analyzing ex vivo methods: (“ex vivo” [All fields] OR “organotypic” [All fields]) AND “retina” [All fields] AND “angiogenesis” [All fields]. The two queries allowed us to find the currently available methods to study retinal angiogenesis, not only *in vitro* studies but also ex vivo studies, including retinal explant models for angiogenesis study.

With this research, after including cross references and excluding duplicates, 625 articles published until May 2017 were found and then judiciously selected.

Articles were screened first by the title and abstract according to the following inclusion criteria: (1) articles written in English or Portuguese; (2) articles related to the theme; and (3) original articles. Based on these criteria, we included experimental articles testing angiogenic or antiangiogenic substances as well as original articles describing new methods or techniques to recreate angiogenesis *in vitro* or ex vivo. As exclusion criteria, we followed the subsequent (1) nonoriginal articles; (2) articles regarding ocular angiogenic pathologies not concerned with the retina; and (3) articles that do not recreate angiogenesis or its steps. Regarding the second exclusion criteria, articles related only to age-related macular degeneration were rejected. Concerning point 3, all the articles that only measured angiogenic factors, such as VEGF or angiopoietins, were rejected. Similarly, all the ex vivo assays that did not evaluate angiogenesis were rejected. After this step, 291 articles were excluded. The remaining 334 articles were selected for full-text reading. On a second level of eligibility, 183 more articles were rejected and 151 studies were selected and included in this review. After these articles' inclusion, relevant data were collected from each paper according to Table 1.

To complement information about the available assays, the fundamentals of the methods, as well as their advantages and disadvantages, were completed with an additional review in the literature. All the articles included in the review can be consulted on Supplementary Material available online at https://doi.org/10.1155/2017/3034953.

## 3. Cell Culture

Most *in vitro* angiogenesis studies depend on cell cultures. Thus, it is important to know which cells can be used, whether it is better to use simple cultures or cocultures and which are the limitations of each cell type.

### 3.1. Endothelial Cells

**Figure 2:**
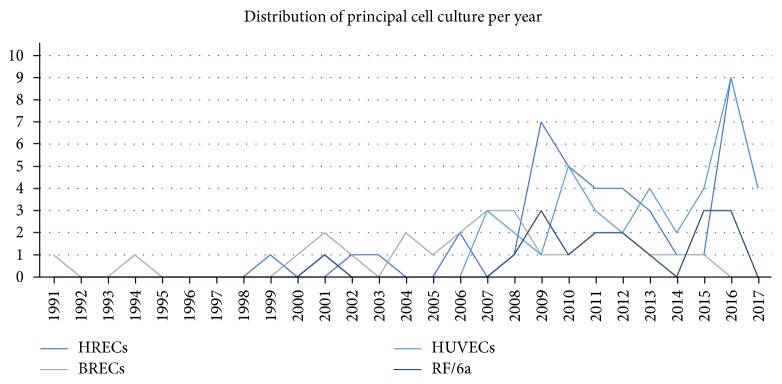
Distribution of principal cell culture per year. BRECs were the most used in the past, being nowadays replaced by human cells such as HRECs and HUVECs. BRECs: bovine retinal endothelial cells; HRECs: human retinal endothelial cells; HUVECs: human umbilical vein endothelial cells; RF/6a: Rhesus monkey choroid and retinal endothelial cells.

ECs are the primary elements of new vessels, and many EC functions are essential requisites for angiogenesis, as previously reported. Therefore, EC cultures are the most used and the most adequate to study retinal angiogenesis. To study angiogenesis, specifically in the retina, it is better to use ECs from retinal tissue, since there are unique properties of ECs obtained from different organs. The most used retinal ECs are bovine retinal endothelial cells (BRECs) [13–37], human retinal endothelial cells (HRECs) [18, 24, 38–77], and Rhesus monkey retinal and choroid endothelial cells (RF/6a) [78–94] (Figure 2). Only one study used Rhesus monkey retinal endothelial cells [95].

However, we also found several studies using nonretinal specific ECs with the aim of studying retinal angiogenesis. In this group, the most used cells are human umbilical vein endothelial cells (HUVECs) [26, 53, 70, 76, 78, 96–130]. Human microvascular endothelial cells (HMVECs) [131, 132] and human dermal microvascular endothelial cells (HMVEC-D) [133] constitute other examples.

It is important to note that some of these cells constitute cell lines (RF/6a) while others constitute primary cultures (BRECs, HRECs, and HUVECs).

Cell lines are transformed, constituting a deviation from normal cells. However, their characteristics are well known, being considered by the scientific community as the standard for research [134]. Furthermore, cell lines are simpler to use and are easier to manipulate for long-term studies. On the other hand, primary cultures represent unchanged cells, being more valid. Nevertheless, they are not well characterized, have limited life-span, proliferate slowly and, generally, require more experiments to validate their origin [135]. Additionally, the cells suffer changes with each passage and behave differently depending on the genetics and age of the individuals from whom they were derived [135]. Despite all the disadvantages, primary cultures are reaching more support since they may be more representative of *in vivo* situation if all the procedures and variables are controlled.

HUVECs are cells derived from the endothelium of veins from human umbilical cord, being one of the most used cells as a laboratory model system. These cells have provided a crucial *in vitro* model for major advances in molecular medicine including mechanisms for the control of angiogenesis or neovascularization in response to hypoxia and inflammation [136]. These vascular cells are easy to be obtained once its source, the umbilical cord, has large vessels, facilitating the experiments using large numbers of cells at early passages [137]. These cells' characteristics may justify their widespread use in the study of retinal angiogenesis. However, the data provided by HUVECs may not accurately reflect the response of the capillary vasculature, which is the site of major pathophysiology [137], thus constituting a limitation for its use. All the studies with HUVECs obtained the cells purchasing them from cell banks. The average number of passages used in angiogenesis assays was 3–5 (minimum 2 [26, 96] and maximum 10 [111]).

**Figure 3:**
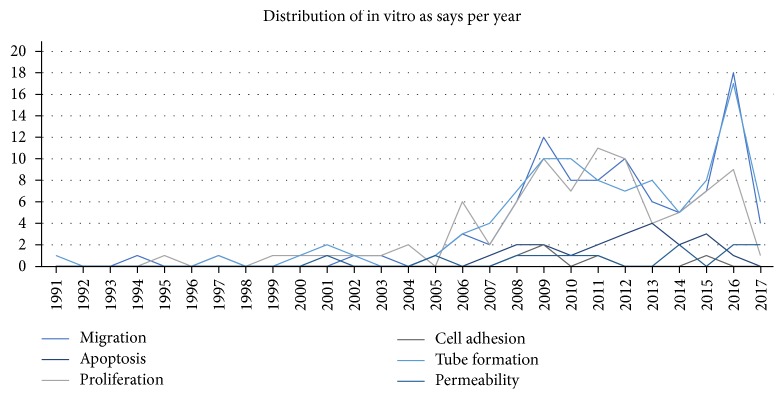
Distribution of *in vitro* assays per year. Progressively, it is visible that there is an increasing use of *in vitro* angiogenesis assays, with a peak in 2016. Migration, proliferation, and tube formation assays are consistently the most applied over time.

Regarding retinal-specific cells, BRECs were the first most used, being currently replaced by a growing use of HRECs (Figure 3). Using BRECs as an *in vitro* system for studying retinal capillary function is mentioned in the literature since the 80s [138, 139]. Thenceforward, several protocols for BREC isolation have been proposed. BRECs are of interest in retinal angiogenesis study since bovine eyes are easy to obtain and manipulate, being available in large quantities [18]. Most of the works isolated BRECs from bovine eyes, using previously described protocols. Only a few authors affirm to have purchased the cells from banks [21, 29]. For the experiments, cells were used from one passage in minimum [32, 33] to 15 in maximum [15], which is important since this is a primary culture and not an immortalized cell line. However, there is one study that uses telomerase-immortalized microvascular ECs from a bovine retina [140].

Despite all the advantages of BRECs, HRECs are always more valid since they have human origin. The highest validity combined with the best isolation techniques that have been developed may justify its increasing use. The need for donors to isolate the cells and consequently a small quantity available to the assays constitutes the major disadvantages. However, nowadays, these cells can also be purchased from cell banks with great quality. Actually, the majority of studies purchased the cells [18, 41, 42, 54, 55].

Other commonly used retinal-specific cells are RF/6a, which is a cell line with retinal and choroidal combined cells. Although the presence of both cells may seem an advantage, it can make it difficult to interpret the real behavior of retinal cells. Furthermore, in the *in vivo* environment, these cells do not contact directly, so their coculture may influence and distort the results. In fact, these cells are different and behave differently. As an example, retinal ECs lack fenestrations to form the blood-retinal barrier (BRB), while choroid ECs have fenestrations with bridging diaphragms [141]. The works that used RF/6a do not mention the number of passages, since this is an immortalized cell line, which also constitutes its main advantage.

In addition, other retinal-specific cells were found to be used in the articles selected, such as retinal ECs from rabbits [14], mice [142–147], and rats [148], as well as primary cultures of newborn porcine neuroretinal ECs [149]. Since these cells are not human in origin, they all exhibit the previously explained drawbacks.

### 3.2. Other Cells

Although most *in vitro* studies only use ECs, other cell types are also important to the angiogenic process, namely, supporting cells such as SMCs, pericytes, fibroblasts, Müller or other glial cells (GCs), and also macrophages.

GCs are neuronal tissue supporters. These cells constitute communicators between vessels and neurons.

Therefore, understanding their behavior in diabetes may provide the necessary clues to interrelate diabetic vasculopathy and neuropathy [150]. From all the GCs, Müller cells were the most studied in retinal angiogenesis assays [19, 20, 25, 48]. Actually, these cells are the principal glia of vertebrate retinas, establishing an anatomical and functional connection between the retinal neurons and the compartments with which they need to interact, such as the retinal blood vessels, the vitreous body, and the subretinal space [151]. Thus, they are the most appropriate cells to coculture with ECs regarding retinal GCs. Other authors evaluated astroglial cells [13, 71, 113]. Astrocytes are also known to play an essential role during retinal vascularization; actually, retinal astrocytic degeneration is associated with failure of the BRB in oxygen-induced retinopathies [152]. Therefore, astroglial cell study is also relevant in angiogenesis retinal assays.

The second most used cell type in combination with ECs are pericytes. Pericytes are perivascular cells that wrap blood capillaries. Thereby, they are clearly important for retinal angiogenesis. Pericyte studies were made in coculture with ECs [37, 153, 154] or independently, regarding proliferation [17].

Some other authors evaluated additional cells related to capillaries, including fibroblasts [99], human vascular SMCs [66], adipose-derived stromal cells (ASCs) [123], and also human placental amniotic membrane-derived mesenchymal stem cells (MSCs) [120], since MSCs are currently being investigated as a treatment for several retinal diseases given their neuroprotective and angiogenic properties [120].

Since we are analyzing retinal angiogenesis, RPE is also widely used by authors to complement the study of EC behavior. RPE secretes essential factors for the structural integrity of the retina, and it is well established that RPE has a crucial role in the development of many retinopathies through the production of many proangiogenic factors [12]. The authors used these cells either in coculture with ECs [88, 129] or independently [45, 59, 70, 81, 96, 109, 116, 119, 124, 125, 155, 156]. When in coculture, it is possible to assess direct cell-cell interactions. In the independent studies, culture media of RPE cells were placed in contact with ECs during angiogenesis assays, intending to evaluate the effects of GF released by RPE.

For biological functions, like angiogenic process, both homotypic and heterotypic cellular interactions are needed. Therefore, coculture models constitute versatile tools for investigating *in vitro* these cellular interactions, allowing to analyze the effects of cell-cell physical contact, secreted factors, and the influence of substrate geometry. On the other hand, coculture results must be complemented by simple culture information since it is difficult to identify the relative contribution of each cell population.

Although neovascular retinopathies are primarily related to hypoxic and metabolic insults, inflammation and innate immune activation contribute significantly to the pathophysiology of these diseases, with a predominant role for macrophages [157]. Indeed, increasingly *in vivo* studies report and evaluate macrophage effect on retinal angiogenesis [158, 159]. Even in ex vivo works, ischemic retinal explant promotes new vessel growth by providing an environment that promotes the survival of ECs and macrophages [160]. *In vitro*, this role is less studied. Among the included papers, only two authors included macrophages in their studies.

Mondragon et al. [95] evaluated apoptosis of ECs exposed to cell media from macrophages treated with high glucose concentrations and low-density lipoprotein (LDL), studying the potential effect of macrophage-secreted factors. Ma et al. [62] studied macrophages independently. Assuming their important role in the pathophysiology of the disease, the authors evaluated macrophage activation by the same factors studied in the assays of EC angiogenesis [62]. A more profound and thorough study of *in vitro* macrophages behavior, either in coculture with endothelial cells or independently, may certainly contribute to a more comprehensive and supported knowledge of neovascular retinopathies.

## 4. *In Vitro* Angiogenesis Assays

Several *in vitro* assays are currently used to study angiogenesis. As previously exposed, angiogenesis *in vitro* assays intend to recreate the several steps of this process.

Regarding the included studies in the research, the most used assays were migration, proliferation, and tube formation of ECs in response to inhibitory or stimulatory compounds. A few articles also studied cell adhesion and permeability while others took apoptosis into account (Figure 3).

In this section, we present the inherent methodologies of each assay, exploring their strong points and drawbacks.

### 4.1. Migration Assays

Migration is one of the first steps needed for angiogenesis. Among the analyzed studies, migration is often taken into account. The most used assays for migration study are Boyden's chamber method assay and “wound-healing assay.”

Boyden's chamber assay was first introduced by Boyden in 1962 for the analysis of leukocyte chemotaxis. The method consists of a chamber of two medium-filled compartments divided by a microporous membrane. During the assay, cells are placed in the upper compartment, being allowed to migrate through the porous membrane to the lower compartment, where chemotactic agents are present. After the incubation period, the membrane is fixed and stained, and the number of cells that have migrated to the lower compartment is counted. Since its first description, this method has been presented with several modifications [161]. Nowadays, Boyden's method is also called filter membrane migration assay, transwell migration assay, or chemotaxis assay [161]. The pores of the membrane can vary between 3 and 14 *μ*m depending on the cell type, to allow cells to deform and migrate rather than passively fall through to the lower side. The most used pore size in the analyzed studies was 8 *μ*m. The separating membrane may be coated with a variety of protein substrates derived from the ECM. Fibronectin was the most frequently used substrate among the analyzed papers.

The second most used assay is the “wound healing assay.” It consists of producing a “wound” in a confluent cell monolayer and captures images at regular intervals during cell migration to close the wound. The images are used to quantify the migration rate of the cells [162]. Some GF stimulate both migration and proliferation. Thus, in this assay, to specifically assess migration, it is useful to add antiproliferative agents to the culture medium. Some authors did not add antiproliferative agents to the culture medium. The ones who did it used preferably 5-fluorouracil at concentrations of 1 mM [32, 163] and 150 ng/mL [143] and porcine serum at 5% in culture medium [34]. All the assays were performed with at least 70% [145] confluent monolayers, but most of the authors used 100% confluence. The quantification of migration was made after 12 hours in minimum [31] and 72 hours in maximum [143], with an average observation time of 24 hours. We found three different ways to quantify migration: (1) measuring the distance moved by the ECs since time 0 after healing; (2) measuring the percentage of uncovered area; and (3) counting the number of cells that migrated beyond a reference line.

To choose the most adequate method for a specific purpose, it is important to know its limitations.

The major advantage of Boyden's chamber method is its high sensitivity to small differences in concentration gradients [164]. Furthermore, it is compatible with adherent and nonadherent cells, it is able to coat the assay surface with a relevant ECM, and it allows to perform chemotaxis assays, as it was found in two of the articles screened [26, 80]. On the other hand, this assay requires many steps, the chemotactic gradient is not linear, it is difficult to maintain transmembrane gradients for prolonged periods, and it is hard to obtain accurate and statistically significant results when only a small number of cells cross through the membrane [165].

The “wound-healing assay” is compatible with any configuration of multiwell assay plate, cells move in a defined direction, and it is possible to visualize cell movement and morphology throughout the experiment. However, there are also some limitations that restrain the validity and comparability of the assays. Indeed, the methods for creating scratches vary between different labs and the size, shape and spacing of the scratches can also vary even in the same lab leading to assay variability. It is also difficult to ensure that control and treatment groups of cells are at the same degree of confluence, and it must be considered that scratches can damage the underlying ECM and cells. Hence, results can be limited by the release of factors from damaged cells. Furthermore, this assay is not suitable for use with nonadherent cells, neither for chemotaxis [165]. Advantages and disadvantages of migration assays are summarized in Table 2.

Some authors use both methods [74, 75, 126]. Although higher costs and more time are required, this approach allows us to compare both results, overtaking some limitations of each one.

### 4.2. Proliferation Assays

EC proliferation is essential for capillary formation. Therefore, proliferation assays are important to study a fundamental step of retinal angiogenesis, being the second most performed assay in the selected papers. EC proliferation can be assessed by direct cell counts, which can be manual or automatized, by quantification of DNA synthesis, metabolic activity, or still by cell surface antigen recognition.

Eleven out of the 87 studies that evaluated proliferation used direct cell counts. The count can be made resorting to a manual method, hemocytometer [17, 48, 64, 142], a semiautomated method [14, 21, 145, 153], or a totally automated cell-count system. The hemocytometer, being a manual method, is prone to more errors, being subject to interuser variation depending on the degree of expertise of the analyst [166]. Furthermore, it is time consuming and requires a high density of cells to be applicable [166]. Its use is more advocated only to confirm and complement other methods, as some authors have done [48, 142]. As an advantage, hemocytometer allows a visual estimation of cell death.

Nowadays, there are several automated cell-count systems available. In general, automated cell-count instruments consist of a digital camera that obtains images and performs analyses through specialized software that requires a minimal user involvement. These methods have several advantages including an orthogonal operating principle, high sensitivity, being able to count a low density of cells, an excellent reproducibility, and high-resolution size information, besides being a nonwasting time method. However, it presents greater variability at higher cell concentrations and has a higher cost [166].

Despite the advantages and disadvantages of each method, a recent study showed that all the three methods are suitable to perform viable cell counts [166].

DNA synthesis can be assessed using radioactive or labelled nucleotide analogues, through 3H-thymidine- and BrdU-based assays. The first method developed to analyze proliferation based on DNA quantification was thymidine incorporation. The need of radioactive facilities and proper waste management in the laboratories constitute its major disadvantages [167]. In our research, we found only seven studies [24, 28, 40, 46, 147, 168, 169] using this method, all before 2011.

Bromodeoxyuridine (5-bromo-2′-deoxyuridine, BrdU, BUdR, BrdUrd) is a synthetic nucleoside analog to thymidine, being incorporated in DNA during the replication process. Using marked anti-BrdU antibodies, the analog incorporation is detected (by immunocytochemistry or by ELISA), being an indicator of cell proliferation. Instead, it is possible to stain the cells with a DNA-binding dye, using then a colorimetric or ELISA reader to quantify it. Concerning limitations, an increase in BrdU staining can only reflect DNA repair while a decrease may be due to cytotoxic rather than cytostatic effects [167, 170].

BrdU incorporation can also be evaluated by cell-cycle analysis with flow cytometric analysis [171]. This method demonstrates the cell-cycle distribution, proliferative state, and the apoptosis rate within a cell population, being able to analyze a large number of cells in a short space of time.

Most of the analyzed studies used commercially available kits to implement proliferation assays based on BrdU incorporation.

Cell proliferation can still be assessed by measuring metabolic activity. Tetrazolium salts or alamar blue are compounds that became reduced (changing the medium color) with metabolic activity of cells. This effect is dependent on the increased activity of the enzyme lactate dehydrogenase (LDH) during proliferation. The absorbance of the media-containing dye solution can be read, through a spectrophotometer or microplate reader, indicating the proliferation rate. The most used tetrazolium salts within the studied articles were 3-(4,5-dimethylthiazol-2-yl)-2,5-diphenyltetrazolium bromide (MTT), 3-(4,5-dimethylthiazol-2-yl)-5-(3-carboxymethoxyphenyl)-2-(4-sulfophenyl)-2H-tetrazolium (MTS), and water-soluble tetrazolium (WST) salts.

Despite the widespread use of MTT, this tetrazolium salt has some limitations, displaying lack of reproducibility, because it is insoluble in standard culture medium. Consequently, MTT assay requires dissolution of formazan crystals in an organic solvent. This can be a problem, since dissolution is not always complete and it can generate bubbles, which interfere with the absorbance readings [172]. In our review, all the studies used the organic solvent dimethyl sulfoxide (DMSO). MTS test overcomes some of these disadvantages, once these salts are soluble. MTS reduction can be accelerated by the addition of an electron-coupling agent, phenazine methosulfate (PMS), which increases test sensitivity. However, the addition of PMS can form crystals in the culture medium, also modifying the absorbance reading [172]. More recent tetrazolium salts are the WST family, namely, WST-1, WST-3, and WST-8. The form WST-8 combines the high stability of WST-1 with the high sensitivity of WST-3 [172]. WST assays are indeed the most used, and we can observe an increase in the WST-8 use instead of WST-1 over time: 15 works [57, 74, 88, 91, 98, 100, 102, 107, 108, 110, 112, 121, 173, 174] used WST-8 since 2007, whereas only 6 studies [39, 54, 104, 113, 140, 175] applied WST-1, having been the last time in 2014. Contrary to tetrazolium salts, only two articles [70, 105] used alamar blue compound to assess metabolic activity, which is surprising since alamar blue has been available for a long time and seems to offer many advantages over tetrazolium salts. Actually, the alamar blue assay provides accurate time-course measurements, has high sensitivity and linearity, involves no cell lysis, is flexible as it can be used with different cell models, is scalable, and can be used with fluorescence and/or absorbance-based instrumentation platforms. Furthermore, it is nontoxic, nonradioactive, and safe for the user and the environment [176].

Cell proliferation can still be assessed by using antigen-based assays that apply antibodies to target antigens present in proliferating cells. Within the analyzed studies, this type of assay was only found in two articles that used ki-67 marker [34, 123].

Regardless of the assay applied, EC proliferation assays are rapid, reproducible, and quantifiable, which may justify its widespread use.

### 4.3. Differentiation Assays

The later stages of angiogenesis (differentiation of ECs) can be assessed through assays that stimulate the formation of capillary-like tubules. The tube formation assays are done by placing ECs onto or into a layer of matrix gel and checking tube formation over time [177, 178]. *In vivo*, ECs are involved by the basement membrane, a thin and highly specialized ECM that maintains the tube-like structures of the blood vessels [177]. ECs placed on basement membrane matrix rapidly attach, align, and form capillary-like tubules [179]. Tube formation on this matrix reasonably simulates *in vivo* situation, having the tight junction formation been confirmed by electron microscopy [178]. Furthermore, the capillary-like structures take up acetylated LDL, which is a marker of differentiation for these cells that is not present in a monolayer culture [177]. The matrix gel composition can vary. However, it is composed mainly of collagen and/or fibrin, stimulating the attachment, migration, and differentiation of ECs into tubule-like structures. Tube formation assays can be two or three dimensional, depending on if the plating of the cells is made on top of a thin layer of ECM or within the matrix [177].

The most frequently used matrix is matrigel, which is a combination of extracellular and basement membrane proteins derived from the Engelbreth-Holm-Swarm (EHS) mouse sarcoma. Matrigel is the most potent matrix for tubule formation. Indeed, tubules begin to form within a few hours [180]. It is a rapid and nonwasting time method, although it can produce overstimulation of ECs [177]. To overcome this limitation, a GF-reduced matrigel was developed, with cytokines and GF levels markedly reduced. Still concerning limitations of matrigel, since it is secreted by EHS mouse sarcoma cells, every batch can be slightly different in terms of quantity of proteins and GF. For this reason, a high variability between results from different lots and manufacturers can be obtained. Furthermore, matrigel may not be adequate for experimentations that require accurate knowledge of all proteins and concentrations [181]. Within the analyzed studies, 53 use the matrigel standard assays, and 18 from the 71 use the GF-reduced matrigel matrix. The reported incubation period varies between 4 hours and 72 hours. The minimal incubation period in standard matrigel was 4 hours [94, 124], while in GF-reduced matrigel was 6 hours [72, 77]. Some studies used different matrices from several angiogenesis assays since nowadays there are several laboratories purchasing angiogenesis kits, all based on the matrix method.

The quantification of tubulogenesis in the analyzed articles is done mainly by quantifying the length of formed capillary-like structures or the number of branch points.

Only 9 [31, 50, 55, 56, 62, 97, 127, 130, 133] apply a 3D model to study tubulogenesis. In these assays, two different methods are reported. The sandwich-type assay uses two layers of matrix with the cells placed between them. The matrix in these assays can be made of matrigel, collagen, fibrin, or a combination. The other method [97, 133] uses a three-dimensional spheroid model of EC differentiation based on Korff and Augustin [182]. It consists of coating microcarrier beads with ECs, then dispersing them throughout the matrix gel, where tubules will form. The dropping of spheroids to the bottom of the gel is a problem with this assay [182].

The biggest advantage of 3D assays is the attempt to more closely simulate the *in vivo* setting. Its general limitation is the difficulty of oxygen and nutrients to diffuse. Furthermore, the quantification of vasculature is also challenging, since it is more likely that one area is not screened, and these assays take more time to run. In the analyzed studies, the sprouting intensity of ECs in spheroid model assay was quantified by an automated image analysis system determining the cumulative sprout length of at least 8 [14] or 10 [97, 127] randomly selected spheroids. In the sandwich method, the number of endothelial sprouts passing the interface from the first to the second layer can be assessed through a phase contrast microscope [31], by counting the number of sprouts [14] or by quantifying cord lengths using computer software [50, 56, 62].

Still about differentiation assays, two papers [18, 32] complemented the results of tube formation assays with a subsequent secondary sprouting assay. In this assay, EC colonies spontaneously survive, proliferate, migrate, and invade the matrix after the original capillary-like tubes have collapsed. Castellon et al. reported that this method appears to be a better indicator of angiogenic potential of various GF than the commonly used tube formation assay [18].

### 4.4. Apoptosis Assays

It is well established that EC apoptosis opposes to neovascularization in the adult organism. Actually, scientific evidence shows that angiogenesis stimulators, such as VEGF or angiopoietin, inhibit EC apoptosis, whereas angiogenesis inhibitors seem to promote apoptosis [183]. Despite its relevance, we only found 23 papers evaluating apoptosis.

There are various ways to evaluate EC apoptosis. Among the studied articles, the most commonly applied assay is based on membrane alterations, resorting to annexin V apoptosis detection kits. Cell surface changes are one of the earlier events of apoptosis: phosphatidylserine (PS) translocates from the inner side of the plasma membrane to the outer layer, becoming PS exposed at the external surface of the cell. Annexin V, a Ca^2+^-dependent phospholipid-binding protein, has high affinity for PS, and fluorochrome-labeled annexin V can be used for the detection of exposed PS through flow cytometry [184].

Terminal deoxynucleotidyl transferase dUTP nick end labeling (TUNEL) assay and caspase monitoring activity assay represent other possibilities for apoptosis study.

DNA fragmentation represents a characteristic hallmark of apoptosis. TUNEL is an established method for detecting DNA fragments. The dUTP can be labeled with various probes to allow detection by light microscopy, fluorescence microscopy, or flow cytometry. The caspases are a family of highly conserved cysteine proteases that play an essential role in apoptosis. Caspase activation can be detected by Western blot, immunoprecipitation, and immunohistochemistry [185].

One study refers to evaluate cell death with no specific method, counting the number of dead cells based on cytomorphological alterations, such as their rounded-up shape, and their phase bright characteristics with cytoplasmic condensation [23]. This is an inexpensive method for the detection of apoptotic cells. Nevertheless, it lacks objectivity, reproducibility, and sensitivity and it is prone to more errors [186].

Regarding the previously mentioned methods, there are also some advantages and disadvantages to consider. The major advantage of annexin V is its high sensitivity, detecting a single apoptotic cell. However, the necrotic cell membranes are also labeled. Therefore, to distinguish apoptotic from necrotic cells, it is necessary to demonstrate membrane integrity of the PS-positive cells. The loss of membrane integrity is a pathognomonic feature of necrosis. Thus, necrotic cells stain with specific membrane-impermeant nucleic acid dyes such as propidium iodide and trypan blue. Contrary, the exclusion of these dyes indicates membrane integrity and as such the presence of apoptotic cells [185].

Regarding caspase monitoring, it is a rapid method and it allows consistent quantification of apoptotic cells. Nevertheless, caspase activation does not necessarily mean that apoptosis will occur and there is significant overlap in the substrate preferences of the members of the caspase family, decreasing the specificity of the assay [185].

Concerning TUNEL assay, it is also very sensitive, rapid, and easily applicable since there are several available kits that can be acquired. Unfortunately, it is expensive, it is not known how many DNA strand breaks are necessary for detection, and it is also subject to false positives from necrotic cells and cells in DNA repair or gene transcription [185]. Advantages and disadvantages of apoptosis assays are summarized in Table 3.

### 4.5. Adhesion Assays

During angiogenesis, cells need to adhere to form new capillaries. Despite this, adhesion assays were only performed by six authors. In these assays, ECs are placed in membrane-containing wells that allow their adherence. In the analyzed articles, fibronectin is the main component of these membranes. After an established time, the wells are washed with phosphate-buffered saline solution to eliminate nonadherent cells. The cells that remain in the wells can be quantified by measuring cellular activity [81, 82, 163], counting the number of cells through an inverted cell culture microscope [187], or using fluorescent markers [21, 117].

### 4.6. Permeability Assays

Vascular permeability is implicated in neovascular retinal diseases. In DR, hyperglycemia is associated with several changes in ECs, among which an increase in vascular permeability [188].

From all the included articles, 13 evaluated permeability. Two techniques to assess EC permeability were found: one based in transwell chambers [16, 21, 29, 33, 64, 71, 75, 103, 154] and another one evaluating transendothelial electrical resistance [29, 33, 36, 73, 140].

Permeability assay with transwell chambers has the same fundamentals as transwell migration assay. The methods differ in the place where the ECs are seeded. In the permeability assay, ECs are placed on the fibronectin-coated membrane and not in the upper chamber. Then a tracer is placed in the upper chamber and allowed to permeate the membrane for a stated time. The quantity of the tracer is measured in the lower chamber, being proportional to EC permeability. The most used tracers in retinal assays are horseradish peroxidase (HRP) [16, 103, 154] and fluorescein isothiocyanate-conjugated dextran [21, 29, 33, 66]. One study used Evans blue as a tracer [71].

Transendothelial electrical resistance is measured through a volt-ohmmeter in a cell monolayer. Decreases in transendothelial electrical resistance across the cell monolayer indicate increases in paracellular permeability [36]. This last technique is faster and more precise; however, it also requires appropriate devices. Both techniques are easy and fast to apply. Thus, implementation of the two methods may reach a more valid result. This combined approach was practiced in two studies [29, 33].

## 5. *Ex Vivo* Angiogenesis Models

Besides cells, organs or parts of organs can also be cultured *in vitro*. Organotypic cultures or ex vivo models maintain the architecture of the tissues closer to the *in vivo* setting, allowing a more accurate assessment of behavior and functions of an organ. Since angiogenesis is a complex process, various ex vivo models have been developed.

Among the articles included, organotypic cultures were also found in some of them. Some of these authors used the classic angiogenesis models: aortic ring assay [36, 72, 77, 115, 117, 163, 169, 189] and chick embryo chorioallantoic membrane assay [83, 117, 118, 148, 190]. However, regarding the specific purpose of studying retinal angiogenesis, they are not the best options since they do not evaluate the intended tissue behavior. Indeed, the main advantage of retinal explant models is its ability to maintain retinal vessel architecture and to assess the contribution that other cell types give to the growth of new vessels [191]. The heterogeneity of vascular endothelium and of its microenvironment requires the use of assay conditions and of endothelial/accessory cell types that most closely resemble the angiogenic disease being studied [4]. Therefore, several descriptions of retinal explant cultures have arisen.

There are specific retinal ex vivo models applied among the selected articles. However, not all intend to evaluate angiogenesis. Actually, most of the found retinal explant protocols aimed at evaluating the toxicity of possible new drugs, some of which antiangiogenic, but they do not evaluate specifically angiogenesis. Instead, they evaluate retinal morphology [74, 130, 192, 193] or perform electroretinograms [194, 195] to assess functionality.

Although these models do not accomplish the primary purpose of this work, they are also extremely important and useful for clinical research of potential antiangiogenic drugs. Indeed, after the evaluation of a drug's effect on angiogenesis, it is necessary to test their toxicity in the tissue.

Specifically concerning the angiogenic process, in our research, we found retinal explant cultures of bovine, human, and mostly mice tissue.

### 5.1. Bovine Retinal Explants

The first description of a retinal explant to study angiogenesis was found in 1990 by Forrester and colleagues [160]. They develop a model system for studying proliferative retinopathy using bovine retinal explants cultured in collagen gels. Cellular outgrowth from retinal explants was evaluated after seven days as single cells from peripapillary explants or as cell sheets and tubular outgrowths from peripheral retinal explants [160].

All the other found bovine retinal explants were only used to evaluate toxicity, as mentioned above.

### 5.2. Human Retinal Explants

In our review, we found a description of a human retinal explant described by Knott et al. in 1999 [191].

Knott and colleagues use human eyes donated for corneal transplant. In this protocol, the used eyes have no history of diabetes and the retinas need to be dissected within 48 hours after death. The obtained explants are placed on the top of a fibrin matrix previously prepared, being then overlaid with a further volume of fibrin matrix. These explants can be incubated and visualized by phase-contrast light microscopy from 1 to 14 days [191]. Some limitations of this protocol concern the age of the donors. Actually, the donors were generally from an older age group. Furthermore, the failure to observe new vessel growth may be due to the extended time between enucleation and sampling of the retinas [191], which is much more difficult to control in human tissues than in other species tissues.

These limitations may justify the fact that there is not any other study in our research applying human retinal explants to study angiogenesis. The difficulty in finding eyes may be another contributor. Actually, retina manipulation requires high expertise, which may not be possible with small samples.

### 5.3. Murine Retinal Explants

More recently, mice are the most used source to implant retinal cultures in order to study angiogenesis.

In 2006, Murakami et al. [196] developed a novel ex vivo system for assessing vitreoretinal angiogenic process that could be observed with time-sequential imaging. The eyes were isolated from 7- to 8-week-old mice, and then the retinas were dissected and cultured for 4 days. The quantification of neovascular sprouts was performed staining the vessels with anti-CD31 (expressed in both quiescent and angiogenic vessels) and type IV collagen (expressed only in mature vessels). Authors also obtained confocal time-sequential images of retinal angiogenesis using a laser scanning microscope. The major advantage of this retinal explant method is the evaluation of vascular sprouting from quiescent and mature vessels in the adult retina, which is much more consistent with the process of pathologic angiogenesis than retinal vascular development in neonates. As a limitation, this ex vivo model does not include blood flow and lacks circulating endothelial progenitors, leukocytes, or hormonal factors, which would play an important role in angiogenesis. Other two selected articles [69, 197] implemented retinal explants based on Murakami et al. [196]. Another work group, Takeuchi et al. [198], applies a similar technique, isolating retinas from mice with the same age and using CD31 to stain the newly formed vessels.

In 2009, DeNiro et al. [175] also used a retinal explant from mice to perform an angiogenesis sprouting assay. In this assay, the retinas are dissected radially into four equal-sized pieces and placed between two layers of matrix of matrigel to produce a sandwich. After different treatments, the cumulative microvessel sprout length per retina was visualized by phase-contrast microscopy and quantified between 1 and 15 days [175].

Later, in 2010, Sawamiphak et al. [199] described a protocol of a culture technique for a neonatal mouse retina that allows the assessment and quantification of acute responses of developing blood vessels to pharmacological manipulation. The technique involves the extraction of the retina from an intact eye and retina flat mounting on a hydrophilic membrane with minimum disturbance of the tissue. The responses of tip endothelial cell sprouting activity to different angiogenic and antiangiogenic factors can be evaluated within 4 hours. This model provides an easily manageable and highly flexible method to evaluate pharmacological manipulations of the developing retinal vessels, allowing highly reproducible quantitative and morphological analysis of the angiogenic responses of ECs, particularly of the extension of filopodial structures in tip ECs, which is the hallmark of the initiation process in active angiogenic sprouting [199]. On the other hand, this method is not suitable for long-term culture to evaluate the overall growth of the vascular bed.

Another ex vivo murine retina angiogenesis assay is described by Rezzola and colleagues [4]. In this protocol, EC sprouts are induced from mature, quiescent retinal vessels of adult mice, similar to the method proposed by Murakami et al. [196]. In Rezzola's method, retinas are isolated from 4- to 5-week-old mice and embedded in fibrin gel, allowing the endothelial sprouting. The sprouts invading the fibrin gel are positively stained by the endothelial marker Bandeiraea simplicifolia BS-I isolectin-B4, thus confirming their identity as endothelial cell sprouts originating from mature retinal vessels. As a limitation, it is not possible to understand the distinct contribution of retinal microvascular arterial or venous endothelium to sprout formation in this assay [4].

Retinal explants from mice were also implemented in two other studies [117, 118] with few differences from the previously exposed methods.

Still concerning mouse retinal explants, in 2016, Amato et al. [200] applied a retinal explant from 4- to 5-week-old mice to produce a model of early DR. For that, retinas were incubated with high glucose environment, hydrogen peroxide (H_2_O_2_), to produce oxidative stress and advanced glycation end (AGE) products. Amato and colleagues do not evaluate angiogenic sprouting. Instead, they evaluate only apoptosis through caspase-3 immunolabeling. However, this may be an interesting method to explore and apply in the study of proliferative retinopathy.

### 5.4. Limitations of *Ex Vivo* Models

In all the procedures, we have to consider some troubleshooting that make ex vivo retinal explants difficult to perform.

First of all, the isolation of the retina is a critical step, requiring a careful handling of the specimen [4]. Second, the age of mice or other animal sources may represent a critical factor to be considered and it is common to observe a significant intersample variability in the angiogenic response of retina fragments [4]. Rezzola and colleagues recommend an adequate period of training of the investigator performing retina fragment isolation to reduce such variability, which may be in part due to erroneous handling of the retina [4].

The expertise requirements to isolate retinas apply to all retinal explants, justifying the still reduced use of retinal explants to study angiogenesis. Concerning the matrix where the sprouting occurs, fibrin matrix seems to be more efficient than collagen matrix [191].

Furthermore, retinal explant methods are not suitable for long-term culture to evaluate the overall growth of the vascular bed. Indeed, the hyperoxic conditions experienced by the retina once extracted from the eye and kept for a long time in culture, as well as the physical disturbance and the lesions made during the retinal explant culture, generally result in regression of the blood vasculature either by increased cell death or by normal vessel pruning. Moreover, the blood circulation that delivers oxygen to the tissue and shapes the developing vascular network is absent [199].

## 6. Discussion

In this review, we made a critical analysis among the *in vitro* and ex vivo assays applied until now to study retinal angiogenesis.

Angiogenesis is a complex process, in which several biological and mechanical factors and structures intervene [1]. Thus, angiogenesis study is not easy and there is no any assay able to recreate completely and accurately this process. The most performed *in vitro* assays regarding retinal angiogenesis are proliferation, migration, and tube formation. In fact, these assays evaluate fundamental steps of angiogenesis, being essential for its knowledge. Among *in vitro* assays, tube formation assay, a differentiation assay, is the one that seems to recreate more closely angiogenesis, since it allows the attachment, migration, and differentiation of endothelial cells into capillary-like structures [177–179]. However, we need to bear in mind that hetero-specific cell interactions are not represented in this method, limiting its validity. Moreover, to completely understand angiogenesis, we should reach all steps separately, which is not possible with tube formation assays. Thereby, differentiation assays can be a more complete type of assay to confirm and support other simpler angiogenic assays, but never a substitute of all of them.

In order to achieve a full understanding of angiogenic process, the maximum possible number of assays should be applied [201].

Within each assay, the choice of the method to apply depends on the laboratories' capacities, as well as the experience of the worker and associated costs. The ideal method should be easy to apply, reproducible, and effective. However, most of the typologies available for each assay allows similar results, with the advantages and disadvantages previously explained.

Another essential discussion point concerning *in vitro* angiogenesis studies is the choice of the cell culture. As previously exposed, it is better to use endothelial cells from retinal tissue, preferably a human retina, once there are specific properties of ECs obtained from different organs and species.

The ex vivo models guarantee a closer relation with the reality *in vivo* [196]. Nevertheless, they do not permit the more sequential and basic approach provided by the other *in vitro* assays. Thus, they must be seen as a complement and not as an alternative to *in vitro* studies. Furthermore, they can be more difficult to implement.

No study using microfluidic systems to study retinal angiogenesis had been found. This kind of system has recently been demonstrated as a potent method for biological studies, including angiogenesis [202]. Therefore, its further application regarding retinal angiogenesis may provide a new way to complement this study.

## 7. Conclusion

In summary, *in vitro* and ex vivo assays are widely used regarding retinal angiogenesis, being easier, more rapid, and more cost-effective than *in vivo* assays. Despite their limitations, these studies constitute, when applied together, a powerful tool to improve angiogenesis knowledge and to study new possible drugs against retinal neovascular pathologies.

## Supplementary Material

Supplementary Table. Articles included in the analysis.

## Figures and Tables

**Table 1:** Information subtracted from the articles. All the included articles were screened according to these points.

Year of publication		
Purpose of the study		
*In vitro* studies?		
Which cells were used?		
Cocultures or independent cultures?
Which angiogenesis assays were performed?		
How many trials?		
How were they performed?		
Resource to kits?		
Was the assay effective?		
Limitations of the assay		
Ex vivo studies?		
Organ used		
How were they implemented?		
Was the assay effective?		
Limitations of the assay		

**Table 2:** Advantages and disadvantages of different migration assays.

	Boyden chamber assay	Wound-healing assay
Advantages	High sensitivity	Allows cell visualization (movement and morphology)
	Compatible with adherent and nonadherent cells	Allows endpoint and kinetic assays
	Allows chemotaxis assays	

Disadvantages	Multistep assay	Variability in scratches dimension
	Chemotactic assay is not linear	Difficult to generate the same confluence degree between groups
	Difficult to maintain transmembrane gradients for prolonged periods	Scratches can damage cells
	Difficult to enumerate cells when their distribution and staining are uneven	Not suitable for chemotaxis
		Not suitable for nonadherent cells

**Table 3:** Advantages and disadvantages of different apoptosis assays.

	Annexin V	Caspase	TUNEL assay
Advantages	High sensitivity	Rapid	High sensitivity
		Allows consistent quantification of apoptotic cells	Rapid
			Easy
Disadvantages	Difficult to differentiate apoptotic from necrotic cells	Low specificity	Expensive
		Activation of caspases does not necessarily mean apoptosis	False positives from necrotic cells, cells in DNA repair or gene transcription
