# Supplementary material for: A Critical Analysis of the Available In Vitro and Ex Vivo Methods to Study Retinal Angiogenesis
Source: J Ophthalmol. 2017 Aug 7;2017:3034953. doi: 10.1155/2017/3034953 (PMC5564124; doi:10.1155/2017/3034953)
Supplement: Supplementary file 1 — Supplementary Table. Articles included in the analysis. [file 3034953.f1.pdf]

ONLINE SUPPLEMENTARY MATERIAL:

Article	Purpose	Cell culture used		Angiogenesis assays applied
		Endothelial cells	Other cells	
**(Forrester et al. 1990)**	To develop a model system for studying proliferative retinopathy using bovine retinal explants			Quantification of neovascular sprouts (*Ex vivo* model of bovine retinal explant)
**(Laterra and Goldstein 1991)**	To evaluate the requirements of RNA and protein synthesis in the generation of neural microvessels	BRECs	Astroglial cells *	Tube formation
**(Koyama et al. 1994)**	To evaluate the autocrine effects of platelet-derived growth factor (PDGF) and their significance on the progression of diabetic retinopathy	BRECs		Migration<br>Proliferation
**(Shima et al. 1995)**	To evaluate the induction of endothelial cells mitogens by hypoxia in retinal cells	Bovine capillary endothelial cells	HRPECs	Proliferation
**(Watanabe et al. 1997)**	To study *in vitro* interactions between cultured endothelial cells and pericytes in order to clarify the mechanism of diabetic proliferative retinopathy	Rabbit retinal endothelial cells	Rabbit retinal pericytes *	Proliferation<br>Tube Formation

**(Grant et al. 1999)**	To test the hypothesis that adenosine regulates angiogenic response of retinal endothelial cells	HRECs		Proliferation
**(Knott et al. 1999)**	To describe a model of human retinal angiogenesis			Quantification of neovascular sprouts (*Ex vivo* model of human retinal explant)
**(Yoshikawa et al. 2000)**	To evaluate the effect of tecogalan sodium on endothelial cells proliferation and *in vitro* angiogenesis	BRECs		Proliferation<br>Tube formation
**(Behzadian et al. 2001)**	To determine transforming growth factor (TGF) beta effects on matrix metalloproteinases (MMPs) as a potential cause of the blood–retinal barrier breakdown at the onset of angiogenesis	BRECs	Glial cells *	Permeability
**(Yan et al. 2001)**	To investigate the potentially synergistic effects of beta fibroblast growth factor (FGF) and VEGF on the proliferation and cord formation of retinal endothelial cells	BRECs	Pericytes	Proliferation<br>Tube formation
**(Gendron et al. 2001)**	To evaluate the possible role of tubedown-1 (tbdn-1) in retinal neovascularization associated with diabetes	RF/6a and HUVECs		Tube formation

**(Castellon et al. 2002)**	To evaluate *in vitro* tenascin-C (TN-C) angiogenic effects on normal and diabetic retinal endothelial cells	BRECs and HRECs		Migration<br>Proliferation<br>Tube formation<br>Secondary sprouting assay
**(Steinle and Granger 2003)**	To determine if nerve growth factor promotes endothelial cell migration and proliferation	HRECs		Migration<br>Proliferation
**(Eichler, Yafai, Wiedemann, et al. 2004)**	To evaluate the effects of müller cells' secretions in retinal endothelial cells behavior	BRECs	Human (MIO-M1) and Guinea-pig müller cells	Proliferation
**(Eichler, Yafai, Keller, et al. 2004)**	To evaluate the effects of hipoxic müller cells secretions on intraocular neovascularization	BRECs	Guinea-pig müller cells	Proliferation
**(Economopoulou et al. 2005)**	To study defensins in hypoxia-induced proliferative retinopathy	BRECs		Migration<br>Tube formation<br>Cell adhesion<br>Permeability
**(Chen et al. 2006)**	To evaluate the effect of blocking ephrin-A (EphA) receptor on VEGF-induced angiogenic responses of cultured retinal endothelial cells	BRECs		Migration<br>Proliferation<br>Tube formation
**(Zhu et al. 2006)**	To investigate the mode of action of protease-activated receptor-2 (PAR2) in retinal angiogenesis	Newborn porcine neuroretinal endothelial cells		Proliferation

**(Wang, Wang, and Sheibani 2006)**	To evaluate the effect of Thrombospondin-1 (TSP1) in migration and proliferation of retinal endothelial cells	Mice retinal endothelial cells	Migration<br>Proliferation
**(Zhang et al. 2006)**	To assess if small interfering RNA (siRNA) may suppress endothelial cells growth and neovascularization by targeting the gene JUN	Murine microvascular endothelial cells	Proliferation<br>Migration<br>Tube formation
**(Coleman et al. 2007)**	To examine the potential of α2(IV) NC1 to regulate retinal microvascular endothelial cells	BRECs	Proliferation<br>Apoptosis<br>Tube formation
**(Xu, Yu, and Duh 2006)**	To investigate the potential function of endothelial cell– derived ADAMTS1, a disintegrin and metalloproteinase with thrombospondin motifs 1 on cell proliferation	HRECs	Proliferation
**(Maines et al. 2006)**	To examine the effects of sphigosine kinase inhibitors on the responses of retinal endothelial cells to VEGF and TNFα	BRECs and HRECs	Proliferation<br>Tube formation

**(Murakami et al. 2006)**	To develop a novel *ex vivo* system for assessing vitreoretinal angiogenic processes that originate from both quiescent and mature vessels			Quantification of neovascular sprouts (*Ex vivo* model of retina from mice)
**(Yafai et al. 2007)**	To study pigment-epithelium derived factor (PEDF) impact on glial–endothelial cellular interactions	BRECs	Retinal guinea pig müller cells	Proliferation
**(Forooghian and Das 2007)**	To compare the *in vitro* anti-angiogenic effects of inhibiting VEGF and hypoxia-inducible factor 1 using ribonucleic acid interference (RNAi)	HUVECs	HRPECs	Tube formation
**(Maier et al. 2007)**	To investigate the effect of JSM6427, an integrin α5β1 inhibiting molecule, on the development of retinal vascular system	HUVECs		Migration<br>Tube formation
**(You et al. 2007)**	To determine the role of Fractalkine (FKN) in ocular angiogenic disorders	HUVECs and BRECs		Migration<br>Tube formation
**(Chikaraishi et al. 2008)**	To examine Rifampicin anti-angiogenic effect on tube formation and proliferation	HUVECs		Proliferation<br>Tube formation

**(Chen et al. 2008)**	To examine the *in vitro* effect of quercetin on choroidal and retinal angiogenesis	RF/6a	Migration<br>Proliferation<br>Apoptosis<br>Tube formation
**(Michaelis et al. 2008)**	To determine the potential role of epoxyeicosatrienoic acids in hypoxia-induced angiogenesis in bovine retinal endothelial cells	BRECs	Migration<br>Tube formation
**(Hata et al. 2008)**	To address the therapeutic potential of fasudil, a potent Rho-kinase inhibitor, in VEGF-elicited angiogenesis	BRECs	Migration<br>Proliferation
**(Kondo et al. 2008)**	To assess the role of bcl-2 in modulation of endothelial cells' proangiogenic phenotype	Retinal endothelial cells from mice	Migration<br>Proliferation<br>Apoptosis<br>Tube formation<br>Cell adhesion<br>Quantification of neovascular sprouts (Aortic ring culture)
**(Navaratna et al. 2008)**	To examine the role of VE-cadherin in cellular processes underlying angiogenesis and the effects of VE-cadherin inhibition on retinal angiogenesis	BRECs	Permeability<br>Migration<br>Proliferation<br>Tube formation

**(Matsunaga et al. 2008)**	To investigate the relationships among VEGFR-1, VEGF, and Pigment epithelium-derived factor (PEDF) in angiogenesis	HUVECs	Fibroblasts *	Tube formation
**(Li et al. 2008)**	To assess the role activin-like kinase receptor 1 (ALK1) plays in neovascularization of VEGF-stimulated HRECs	HRECs		Migration<br>Proliferation<br>Tube formation
**(Jiang et al. 2009)**	To examine the effects of very-low-density-lipoprotein receptor (VLDLR) on the angiogenic functions of retinal vascular endothelial cells	Mouse Retinal Vascular Endothelial Cells		Migration<br>Proliferation<br>Tube formation
**(Matsunaga et al. 2010)**	To test Vaccinium myrtillus (Bilberry) extracts effects on angiogenesis	HUVECs		Migration<br>Proliferation<br>Tube formation
**(You et al. 2009)**	To investigate the role of Cysteine-rich 61 (Cyr61/CCN1) in ocular angiogenesis and proliferative diabetic retinopathy	RF/6a		Migration<br>Proliferation<br>Tube formation
**(Cano Mdel et al. 2009)**	To evaluate the antiangiogenic potential of an 18-mer peptide derived from type 1 thrombospondin repeat-containing protein WISP-1 (wispostatin-1)	HRECs		Migration

**(Basu et al. 2009)**	To investigate the role of the serine proteinase inhibitor PAI-1 in facilitating retinal angiogenesis	HRECs		Migration
**(Boosani et al. 2009)**	To determine the impact of the antiangiogenic factor α1(IV)NC1 on VEGF-mediated proangiogenic activity in mouse retinal endothelial cells	Primary Mouse retinal endothelial cells		Migration<br>Proliferation<br>Apoptosis<br>Tube formation
**(Kim, Lee, et al. 2009)**	To evaluate rosmarinic acid effects on retinal endothelial cells	HRECs		Proliferation<br>Tube formation
**(Huang et al. 2009)**	To investigate the effect of Robo4 in choroid–retina endothelial cells and human retinal pigment epithelial cells	RF/6a	HRPECs	Migration<br>Proliferation<br>Tube formation<br>Cell attachment assay
**(Zhao et al. 2009)**	To evaluate the effect of KV11, a novel 11-mer peptide from human apolipoprotein, against retinal neovascularization	BRECs		Migration<br>Proliferation

**(DeNiro, Alsmadi, and Al-Mohanna 2009)**	To evaluate the effects of YC-1, a HIF-1 inhibitor, on the morphological, biochemical and molecular changes in human retinal microvascular endothelial cells	HRECs		Migration<br>Proliferation<br>Apoptosis<br>Tube formation
**(Afzal et al. 2010)**	To evaluate if carboxyamidotriazole could have anti-angiogenic effects on ocular angiogenesis models	HRECs, Human dermal endothelial cells	HRPECs	Proliferation<br>Tube formation
**(Kim, Kim, Oh, et al. 2009)**	To investigate the antiangiogenic properties of *N*-hydroxy-7-(2-naphthylthio) heptanomide (HNHA)	HUVECs		Migration<br>Proliferation<br>Tube formation
**(Kim, Kim, Lee, et al. 2009)**	To evaluate the inhibitory effect of decursin on retinal neovascularization	HRECs		Migration<br>Proliferation<br>Tube formation
**(Nakamura et al. 2010)**	To assess the efficacy of ruboxistaurin as an anti-angiogenic agent	HUVECs		Migration<br>Proliferation<br>Tube formation
**(Ma et al. 2009)**	To determine if an orally available form of calpain inhibitor, SNJ-1945, prevent angiogenesis induced by VEGF in cultured retinal endothelial cells	HRECs		Migration<br>Tube formation

**(Yanni et al. 2010)**	To investigate the ability of amfenac to inhibit discrete aspects of the angiogenic cascade *in vitro*	HRECs	Rat retinal müller cells	Proliferation<br>Tube formation
**(Lara-Castillo et al. 2009)**	To explore the role of ANP in VEGF-mediated retinal vascular leakage and angiogenesis	HRECs		Permeability
**(Huang et al. 2009)**	To investigate the function of Robo1 and its possible role in retinal angiogenesis	RF/6a		Migration<br>Proliferation<br>Tube formation<br>Cell attachment assay
**(Unoki, Murakami, Ogino, et al. 2010)**	To elucidate the effects of anecortave desacetate treatment on the kinetics of neovascular sprouting and its molecular mechanisms in retinal explants of mice			Quantification of neovascular sprouts (*Ex vivo* model of retina from mice)
**(Tang et al. 2010)**	To evaluate CYP1B1 effects on angiogenesis	Mice retinal endothelial cells		Migration<br>Tube formation
**(Unoki, Murakami, Nishijima, et al. 2010)**	To investigate how SDF-1 and its receptor, CXCR4, influence neovascular sprouting	HRECs		Quantification of neovascular sprouts (*Ex vivo* model of retina from mice)
**(Magnussen et al. 2010)**	To investigate the antiangiogenic activity of VEGF165b and its effect on retinal epithelial and endothelial cell survival	Human microvascular endothelial cells (HMVECs), HUVECs, HRECs	HRPE cells, and ARPE-19 cells	Migration

**(Pourgholami et al. 2010)**	To investigate the antiangiogenic effect of albendazole on non-cancerous models of angiogenesis	HUVECs	Permeability<br>Migration<br>Proliferation<br>Tube formation
**(Matesanz et al. 2010)**	To investigate the effect of -3 polyunsaturated fatty acids (PUFAs) on angiogenic signaling in retinal microvascular endothelial cells	BRECs	Migration<br>Proliferation<br>Apoptosis<br>Tube formation
**(Hoang, Smith, and Senger 2011)**	To evaluate the effects of Calpain inhibitors in cappilaries organization	HRECs	Tube formation
**(Hoang, Smith, and Senger 2010)**	To investigate the inhibition of glycogen synthase kinase-3β (GSK-3β) as a means for improving the architecture and functionality of pathological blood vessels in the retina	HRECs	Tube formation
**(Sawamiphak, Ritter, and Acker-Palmer 2010)**	This protocol details a culture technique for neonatal mouse retina that allows the assessment and quantification of acute responses of developing blood vessels to pharmacological manipulation		Quantification of neovascular sprouts (*Ex vivo* model of retina from mice)

**(Jiang et al. 2011)**	To examine the potential role of APE1/Ref-1 for inhibiting retinal angiogenesis	Human umbilical cord blood-derived endothelial colony forming cells (ECFCs)<br>Mouse retinal vascular endothelial cells	Mouse retinal pericytes	Migration<br>Apoptosis<br>Proliferation<br>Tube formation
**(Nishiguchi et al. 2010)**	To investigate the role of soluble Heparan-sulfane/heparin GAGs on retinal angiogenesis	HUVECs		Migration<br>Proliferation<br>Tube formation
**(Xu et al. 2010)**	To study the antiangiogenic activity of two small peptides (H-RN and H-FT) derived from the hepatocyte growth factor kringle 1 domain (HGF K1) in angiogenesis	RF/6a		Migration<br>Proliferation<br>Tube formation<br>Quantification of neovascular sprouts (Chick chorioallantoic membrane assay)
**(Kim et al. 2011)**	To investigate whether gold nanoparticle (GNP) can inhibit retinal neovascularization	HRECs		Migration<br>Proliferation<br>Tube formation<br>Cell viability
**(Hasan et al. 2011)**	To assess the effect of CCN family member 1 (CCN1)/ Cysteine-rich angiogenic inducer 61(Cyr61) on angiogenesis	Rat retinal endothelial cells		Migration<br>Proliferation<br>Cell Adhesion

**(Rymo et al. 2011)**	To evaluate microglia's effect on vessel sprouting in the aortic ring culture			Quantification of neovascular sprouts (Aortic ring assay)
**(Nakamura et al. 2011)**	To identify the role of tissue kallikrein in retinal neovascularization	HUVECs<br>HRECs		Migration<br>Proliferation<br>Tube Formation
**(Banumathi et al. 2011)**	To investigate *in vitro* role of Ca^{2+}-dependent signaling in VEGF-induced angiogenesis in the retina	BRECs		Migration<br>Proliferation<br>Tube formation<br>Secondary sprouting assay
**(He et al. 2011)**	To evaluate the role of delta-like ligand 4 (DLL4) in neovascularization	RF/6a		Migration<br>Proliferation<br>Tube formation
**(Sun et al. 2011)**	To explore the potential role of insulin-like growth factor binding protein-related protein 1 (IGFBPrP1) as an endogenous angiogenesis inhibitor in the prevention of VEGF-induced retinal angiogenesis	RF/6a		Migration<br>Proliferation<br>Apoptosis<br>Tube formation
**(Yu, Liang, and Ferrara 2011)**	To compare different protein VEGF inhibitors for their ability to inhibit VEGF-stimulated activities	HUVECs		Migration<br>Proliferation
**(Zaniolo et al. 2011)**	To explore the role of ghrelin and its growth hormone secretagogue receptor 1a (GHSR-1a) in proliferative retinopathy	Porcine brain microvascular endothelial cells	Rat muller cells<br>Astrocytes	Proliferation

				Quantification of neovascular sprouts (Aortic explants microvascular sprouting)
**(Cai et al. 2011)**	To assess *in vitro* vascular permeability by measuring transendothelial resistance and paracellular permeability to dextran	BRMECs		Permeability
**(Jhanji et al. 2011)**	To explore the antiangiogenic property of isoliquiritigenin			Quantification of neovascular sprouts (Chick choriollantoic assay)
**(Stewart et al. 2011)**	To compare the characteristics of primary cultures of isolated human choroidal endothelial cells (hCEC) and retinal endothelial cells (hREC), and their proliferation responses to stimulation with VEGF 121 and 165, comparing the anti-proliferative effects of these drugs	HRECs and HCECs		Proliferation
**(Kumar et al. 2011)**	To elucidate the mechanism of interaction between human retinal progenitor cells and HUVECs that modulates neovascularization response	HUVECs	Human retinal progenitor cells *	Tube formation
**(Guduric-Fuchs et al. 2012)**	To identify and quantify microRNAs and other small regulatory non-coding RNAs (ncRNAs) which may regulate angiogenesis	BRECs		Migration<br>Proliferation<br>Tube formation

**(Jadhao et al. 2012)**	To assess if nerve growth factor may stimulate CD34+ endothelial progenitor cells to convert to an angiogenic phenotype	HRECs	CD34 cells*	Migration<br>Proliferation<br>Tube formation
**(Bai et al. 2012)**	To investigate PEGylated-PEDF long-term effects on preventing angiogenesis	HUVECs		Migration<br>Proliferation<br>Apoptosis<br>Tube formation
**(Xu et al. 2012)**	To explore the role of MEF2C in retinal vascularization during normal development	HRECs		Migration<br>Tube formation<br>Apoptosis
**(Deissler, Deissler, and Lang 2012)**	To investigate the effects of bevacizumab on VEGF-induced changes of iBREC properties and potential uptake and accumulation of both inhibitors	Telomerase-immortalised microvascular endothelial cells from bovine retina (iBREC)		Permeability<br>Migration<br>Proliferation
**(Nakamura et al. 2012)**	To study *in vitro* proliferation and migration of human retinal microvascular endothelial cells induced by VEGF-A	HRECs		Migration<br>Proliferation
**(Luo, Wu, and Gu 2012)**	To investigate the effects of a modified Dahuang Zhechong Pill (MDZP) on the angiogenesis of rhesus choroid-retina endothelial cells and its preliminary mechanism	RF/6a		Migration<br>Proliferation<br>Tube formation

**(Giddabasappa et al. 2012)**	To evaluate the *in vitro* anti-angiogenic effects of estrogen-receptor beta (ERb) selective agonist, b-LGND2, using human retinal microvascular endothelial cell cultures	HRECs		Migration<br>Proliferation<br>Apoptosis<br>Tube formation
**(Zheng, Gu, and Xu 2012)**	To evaluate the effect of ZY1, a novel peptide, against ocular neovascularization	RF/6a		Migration<br>Proliferation<br>Tube formation
**(Lu et al. 2012)**	To investigate the antiangiogenic activity of Kringle 1 Domain of Human Hepatocyte Growth Factor (HGFK1)	HUVECs		Migration<br>Proliferation
**(Yan et al. 2012)**	To investigate whether 15-Lipoxygenase-1 (15-LOX-1) plays an important role in the regulation of angiogenesis, inhibiting hypoxia-induced proliferation of retinal microvascular endothelial cells and the underlying mechanism	Primary mice Retinal Microvascular Endothelial Cells		Proliferation
**(Yokouchi et al. 2013)**	To investigate whether retinal pigment epithelial cells produce pro-angiogenic factors under high glucose conditions in vitro.	HRECs	HRPECs (ARPE-19)	Tube formation

**(Liu et al. 2013)**	To assess whether activation of the unfolded protein response (UPR) and downstream Alpha-crystallin B chain (CRYAB) up regulation may sustain the VEGF signaling pathway and if targeting both extracellular VEGF and the UPR will represent a more effective strategy than current extracellular VEGF therapies alone	BRECs		Tube formation
**(Morales et al. 2013)**	To determine whether epithelial membrane protein 2 (EMP2) regulates VEGF expression in the RPE cell line, ARPE-19, and to watch if that influences HUVECs behavior	HUVECs	APRE-19	Migration<br>Tube formation
**(Bai et al. 2013)**	To investigate the efficacy and potential mechanisms of endostatin for the prevention of retinal neovascularization	HUVECs		Migration<br>Proliferation<br>Apoptosis<br>Tube formation
**(Buehler et al. 2013)**	To evaluate the anti-angiogenic effects of semaforins	HRECs		Tube formation
**(Rezzola et al. 2013)**	To describe a new *ex vivo* murine retina angiogenesis assay			Quantification of neovascular sprouts (Retinal explant of mice)
**(Wang et al. 2013)**	To examine the inhibitory effects of Conbercept (KH902) on angiogenesis	HUVECs		Migration<br>Proliferation<br>Apoptosis<br>Tube formation

**(Yu et al. 2013)**	To identify the role of Semaphorin 3A (Sema3A) in retinal neovascularization	HUVECs		Migration<br>Proliferation<br>Apoptosis<br>Tube formation
**(Shmueli et al. 2013)**	To investigate serpin-derived-peptide activity in human retinal endothelial cells	HRECs		Migration<br>Apoptosis
**(Du et al. 2013)**	To investigate whether the overexpression of decorin in retinal pigmented epithelial cells under hypoxia alters the *in vitro* angiogenic ability of co-cultured choroid-retinal endothelial cells and to explore the possible mechanisms involved	RF/6a	ARPE-19*	Migration<br>Proliferation<br>Tube formation<br>Cell viability
**(Zhang et al. 2014)**	To investigate the effects of VEGF-B on proliferation and migration in EA.Hy926 cells.	EA.Hy926 cells		Migration<br>Proliferation
**(Jo et al. 2014)**	To investigate the therapeutic potential and safety profiles of high affinity peptides targeting VEGF which are identified using an 'aptide' technology	HUVECs		Migration<br>Proliferation<br>Tube formation<br>Cell viability
**(Takeuchi et al. 2014)**	To investigate the role of epidermal growth factor-like domain 7 (EGFL7) in VEGF driven angiogenesis using an *ex vivo* Matrigel-embedded mouse eye cup assay			Quantification of neovascular sprouts (Retinal explants from mice)

**(Xu et al. 2014)**	To investigate the effect of endothelial glycolysis on angiogenesis	HUVECs		Proliferation<br>Tube formation
**(Jittiporn et al. 2014)**	To evaluate the anti-angiogenic effects of α-mangostin in relation to ROS formation on bovine retinal endothelial cells	BRECs		Permeability<br>Migration<br>Proliferation<br>Tube formation<br>Quantification of neovascular sprouts (Aortic ring assay)
**(Ghim et al. 2014)**	To investigate the potential roles of Phospholipase D2 (PLD2) in endothelial cells under hypoxia	Primary mouse lung endothelial cells<br>HUVECs		Migration<br>Quantification of neovascular sprouts (Aortic ring assay)
**(Ma et al. 2014)**	To determine Phospholipase D2 (PLD2) involvement in survival,<br>migration, and sprouting of endothelial cells under hypoxic conditions	HRECs	Macrophages	Proliferation<br>Apoptosis<br>Tube formation
**(Zhang et al. 2015)**	To examine the effects of neurotrophin receptor p75 (p75NTR) in hypoxia-induced, angiogenesis-related factors in retinal pigmented epithelium	HUVECs	ARPE-19	Proliferation<br>Apoptosis<br>Tube formation
**(Beltramo et al. 2014)**	To assess the behavior of retinal pericytes cultured in physiological and diabetic-like conditions (high glucose and/or hypoxia)	Human microvascular endothelial cells	Human retinal pericytes *	Permeability<br>Migration<br>Apoptosis<br>Tube formation

**(Mondragon et al. 2015)**	To evaluate the response of retinal capillary endothelial cells to macrophage-derived transforming growth factor beta (TGFβ) and to the BIGH3 protein	Rhesus monkey retinal endothelial cells	Macrophages	Apoptosis
**(Wang et al. 2015)**	To confirm the inhibition of advanced glycation end products (AGE)-induced angiogenesis in retinal endothelial cells by DIIV and to investigate the potential underlying mechanisms	RF/6a		Migration<br>Proliferation<br>Tube formation
**(Yan et al. 2015)**	To elucidate whether lncRNA-myocardial infarction–associated transcript (MIAT) is involved in diabetes mellitus–induced microvascular dysfunction	RF/6a		Migration<br>Proliferation<br>Tube Formation
**(Li et al. 2015)**	To investigate the effects of quercetin on VEGF-induced *in vitro* choroidal and retinal angiogenesis	RF/6a		Migration<br>Proliferation<br>Tube formation
**(Rezzola et al. 2015)**	To evaluate the effect of Escherichia coli polysaccharide K5 [K5-N,OS(H)] as a multitarget molecule inhibiting VEGF-driven angiogenic responses on different *in vitro* and *ex vivo* assays	HUVEC		Migration<br>Proliferation<br>Cell adhesion<br>Quantification of neovascular sprouts (Aortic ring assay, choriollantoic membrane assay and mice retinal explants)

**(Dal Monte et al. 2015)**	To investigate the effectiveness of UPARANT, a urokinase receptor-derived peptide inhibitor of VEGF, in counteracting pathologic neovascularization in the retina	HUVECs		Migration<br>Quantification of neovascular sprouts (choriollantoic membrane assay and retinal explants)
**(Kim et al. 2015)**	To evaluate *in vitro* angiogenesis in VEGF- or hypoxia-stimulated endothelial and retinal cells	HUVECs	ARPE-19	Tube formation
**(Durham et al. 2015)**	To establish the regulatory roles that pericytes have in coordinating retinal endothelial cell growth and angiogenic potential	BRECs	Human Pericytes *	Proliferation<br>Tube formation
**(Basavarajappa et al. 2015)**	To study cell-based structure–activity relationship in order to develop more potent cremastranone analogues, with improved anti-proliferative selectivity for retinal endothelial cells	HRECs		Migration<br>Proliferation<br>Cell viability<br>Apoptosis<br>Tube formation
**(Kim et al. 2016)**	To evaluate the role of systemically injected human placental amniotic membrane derived on pathological neovascularization of proliferative retinopathy	HUVECs	Human placental amniotic membrane derived mesenchymal stem cells *	Proliferation
**(Zhu et al. 2016)**	To study the role of interleukin-17A in angiogenesis	HUVECs		Proliferation<br>Tube formation

**(Johnen et al. 2015)**	To evaluate the effect of recombinant PEDF (rPEDF) in ocular neovascularization	HUVECs	Rat retinal pigmented epitelial cells<br>ARPE-19	Migration<br>Apoptosis<br>Tube Formation
**(Madonna et al. 2016)**	To test the hypothesis that glucose-induced hyperosmolarity, occurring in diabetic hyperglycemia, promotes retinal angiogenesis, and that interference with osmolarity signaling ameliorates excessive angiogenesis and retinopathy	Human aortic endothelial cells and Human Microvascular endothelial cells		Migration<br>Tube formation
**(LeBlanc et al. 2016)**	To examine the role of Hepatoma-derived growth factor in regulating ocular vasculature	HRECs		Permeability<br>Migration<br>Proliferation
**(Yamaguchi et al. 2016)**	To investigate the therapeutic potential of a Rho-associated coiledcoil– containing protein kinase (ROCK) inhibitor ripasudil (K-115) on retinal neovascularization and hypoxia	HRECs		Migration
**(Kobayashi et al. 2016)**	To investigate which role tenascin-C plays in angiogenesis	HRECs	Human vascular smooth muscle cells	Migration<br>Proliferation<br>Tube Formation

**(Li et al. 2016)**	To evaluate the anti-angiogenic profile of a novel fusion protein, Tat PTD-Endostatin-RGD, to treat retinal neovascularization	EAHY926 endothelial cells	Migration<br>Tube formation<br>Quantification of neovascular sprouts (Chick embryo chorioallantoic membrane assay)
**(Li, Du, and Chang 2016)**	To investigate the effects of autophagy on *in vitro* hypoxia-induced choroidal and retinal angiogenesis	RF/6a	Migration<br>Tube formation<br>Cell viability
**(Abu El-Asrar et al. 2016)**	To assess matrix metalloproteinase-1 (MMP-1) effect on *in vitro* cell migration angiogenesis	HRECs	Migration
**(Amato et al. 2016)**	To describe an *ex vivo* model of early diabetic retinopathy		Quantification of neovascular sprouts (Ex vivo retinal mouse explant)
**(Zhou et al. 2016)**	To determine whether interleukin (IL)-12 plays a role in the neovascularization	HRECs	Tube formation
**(Siemerink et al. 2016)**	To investigate the role of CD34 in angiogenesis using *in vitro* angiogenesis models in the presence or absence of CD34-specific small interfering RNA (siRNA)	Immortalized human microvascular dermal endothelial cells (HMEc-1)	Migration<br>Tube formation

**(Gong, Fu, et al. 2016)**	To evaluate if the inhibition of CYP2C activity will add to the protective effects of ω-3 LCPUFA on neovascular eye diseases	HRECs		Migration<br>Tube Formation<br>Aortic ring assay
**(Lee, Lee, et al. 2016)**	To evaluate the effect of an extract of Cnidium officinale Makino and its bioactive compound, butylidenephthalide, on the migration and tube formation of human umbilical vein endothelial cells	HUVECs		Migration<br>Tube formation
**(Hajmousa et al. 2016)**	To evaluate the adipose-derived stromal cells effect in diabetic retinopathy	HUVECs	Adipose-derived stromal cells	Proliferation<br>Apoptosis
**(Lee, Sun, et al. 2016)**	To clarify the effect of SH-11037 (an homoisoflavonoid analogue) in retinal angiogenesis	HUVECs, HRECs	ARP-19	Proliferation

**(Wang et al. 2016)**	To investigate the potential signal mechanism of tissue factor (TF) in the regulation of the expression of VEGF in human retinal pigment epithelial (ARPE-19) cells	RF/6a	ARP-19*	Migration<br>Proliferation<br>Tube Formation
**(Yun et al. 2017)**	To evaluate the effect of STAT 3 in diabetic retinopathy	HRECs	Human brain astrocytes<br>Human brain pericytes<br>HMO6 (human microglial cell line)	Permeability
**(Yu et al. 2016)**	To investigate the inhibitory mechanism of erianin on retinal neoangiogenesis and its contribution to the amelioration of diabetic retinopathy	RF/6a	Microglia cell line *	Migration<br>Tube Formation
**(Spuul et al. 2016)**	To analyze the effect of podosomes in retinal sprouting angiogenesis	Human Microvascular Endothelial Cells		Migration

**(Gong, Shao, et al. 2016)**	To evaluate the effect of fenofibrate in retinal angiogenesis	HRECs		Migration<br>Tube Formation<br>Quantification of neovascular sprouts (Aortic ring assay)
**(Xu et al. 2016)**	To study the effects of down syndrome critical region gene 1 (DSCR1) on retinal angiogenesis	Mouse retinal microvascular endothelial cells	Primary retinal ganglion cells	Proliferation<br>Migration<br>Tube formation
**(Yiu et al. 2016)**	To employ type II clustered regularly interspaced short palindromic repeats (CRISPR)-Cas9 endonuclease to suppress ocular angiogenesis by genomic disruption of VEGF-A in human RPE cells	HUVECs	ARP-19	Tube Formation
**(Motta et al. 2016)**	To investigate the molecular mechanisms of the antiangiogenic activity of UPARANT, an antagonist of the urokinase-type plasminogen activator receptor (uPAR), on primary human retinal endothelial cells (HREC) as a model of *in vitro* angiogenesis	HRECs		Migration<br>Proliferation<br>Tube Formation<br>Permeability

**(Guo et al. 2016)**	To evaluate the effect of 4-Hydroxy-7-oxo-5-heptenoic Acid (HOHA)-lactone on ocular neovascularization	HUVECs	ARP-19<br>Human primary retinal pigmented epithelial cells	Proliferation<br>Migration<br>Tube Formation
**(Xiong et al. 2016)**	To elucidate the role of insulin gene enhancer protein ISL-1 (Islet-1) in angiogenesis and regulation of VEGF expression *in vitro*	HUVECs		Proliferation<br>Migration<br>Tube Formation
**(Chen et al. 2017)**	To investigate the role of EP3 in retinal angiogenesis and evaluate the underlying mechanisms	HUVECs		Tube Formation
**(Xu et al. 2017)**	To investigate the anti-angiogenic effect of kaempferol and explore its underlying molecular mechanisms under diabetic-like environment	HRECs		Proliferation<br>Migration<br>Tube formation

**(Xie et al. 2017)**	To explore the mechanism of transcriptional regulation of Roundabout Guidance Receptor 4 (Robo4) in retinal endothelial cells, and investigate the effects of this regulation on cellular functions under hyperglycemic conditions	HRECs		Migration<br>Permeability<br>Tube Formation
**(Bucher et al. 2017)**	To evaluate the effect of antibody-mediated inhibition of tspan12 on vasoproliferative retinopathy	HUVECs		Migration<br>Tube formation
**(Lam et al. 2017)**	To identify the role of RUNX1 in retinal angiogenesis	HRECs, HUVECs		Migration<br>Tube formation
**(Spencer et al. 2017)**	To analyze the synergistic interaction between endothelial cells and retinal pigment epithelium	HUVECs	ARP-19*	Tube Formation

Table 4. Articles included in the analysis

*in co-culture with endothelial cells

BRECs: Bovine Retinal Endothelial Cells; HRECs: Human Retinal Endothelial Cells; HRPECs: Human Retinal Pigmented Epithelium Cells; HUVECs: Human Umbilical Vein Endothelial Cells; RF/6a: Rhesus Monkey Choroid and Retina Endothelial Cells; ARP-19: immortalized cell line of human retinal pigment epithelium; VEGF: vascular endothelial growth factor; HRPECs: Human Retinal Pigmented Epithelial Cells.

The referred purposes are the study's purposes related to the review. It does not mean the study does not have other aims, just as it also does not mean that other cell lines or assays could be applied.
